# T Cell-Dependence of Lassa Fever Pathogenesis

**DOI:** 10.1371/journal.ppat.1000836

**Published:** 2010-03-26

**Authors:** Lukas Flatz, Toni Rieger, Doron Merkler, Andreas Bergthaler, Tommy Regen, Mariann Schedensack, Lukas Bestmann, Admar Verschoor, Mario Kreutzfeldt, Wolfgang Brück, Uwe-Karsten Hanisch, Stephan Günther, Daniel D. Pinschewer

**Affiliations:** 1 Department of Pathology and Immunology, University of Geneva, Geneva, Switzerland; 2 Institute of Experimental Immunology, Department of Pathology, University Hospital of Zurich, Zurich, Switzerland; 3 Department of Virology, Bernhard-Nocht-Institute for Tropical Medicine, Hamburg, Germany; 4 Department of Neuropathology, Georg-August-University, Göttingen, Germany; 5 Institute of Clinical Chemistry, University Hospital of Zurich, Zurich, Switzerland; 6 Unilabs Dr. Weber, St. Gallen, Switzerland; 7 W.H.O. Collaborating Center for Neonatal Vaccinology, University of Geneva, Geneva, Switzerland; University of California San Francisco, United States of America

## Abstract

Lassa virus (LASV), the causative agent of Lassa fever (LF), is endemic in West Africa, accounting for substantial morbidity and mortality. In spite of ongoing research efforts, LF pathogenesis and mechanisms of LASV immune control remain poorly understood. While normal laboratory mice are resistant to LASV, we report that mice expressing humanized instead of murine MHC class I (MHC-I) failed to control LASV infection and develop severe LF. Infection of MHC-I knockout mice confirmed a key role for MHC-I-restricted T cell responses in controlling LASV. Intriguingly we found that T cell depletion in LASV-infected HHD mice prevented disease, irrespective of high-level viremia. Widespread activation of monocyte/macrophage lineage cells, manifest through inducible NO synthase expression, and elevated IL-12p40 serum levels indicated a systemic inflammatory condition. The absence of extensive monocyte/macrophage activation in T cell-depleted mice suggested that T cell responses contribute to deleterious innate inflammatory reactions and LF pathogenesis. Our observations in mice indicate a dual role for T cells, not only protecting from LASV, but also enhancing LF pathogenesis. The possibility of T cell-driven enhancement and immunopathogenesis should be given consideration in future LF vaccine development.

## Author Summary

Lassa virus (LASV) is the causative agent of Lassa fever (LF), accounting for substantial morbidity and mortality in West Africa. Yet the mechanisms leading to disease remain poorly understood. Here we propose a concept whereby the body's immune defense either defeats LASV rapidly or, if unsuccessful, becomes an essential facilitator of disease. This latter paradoxical postulate stems from observations in genetically engineered (HHD) mice, which we found to be susceptible to LF. HHD mice differ from resistant wild type mice in that they have a humanized repertoire of T cells, a main component of the mammalian immune system. Counterintuitively, we could protect HHD mice against LF by experimentally removing their T cells. We further found that LF correlated with widespread activation of macrophages, which again depended on T cells. Similar to T cells, macrophages are important players in our body's defense system, but their inflammatory products are also candidate mediators of LF. Taken together, these findings suggest that LF may represent an inappropriate host response to infection. Specifically, our study demonstrates a two-faced role of T cell responses against LASV. Such detrimental aspects of immune defense need to be given consideration in future LF vaccine development, to avoid enhancement of disease in vaccinated individuals.

## Introduction

Lassa virus (LASV) is the causative agent of Lassa fever (LF) [1]. It accounts for an estimated number of 300′000 infections and several thousand deaths in endemic areas each year [2], while imported cases have been reported from around the globe [3]. The virus is listed category A by the Center for Disease Control and Prevention [4]. So far, LASV vaccines have remained unavailable for clinical use, and Ribavirin, the only available therapy, has shown limited efficacy [5]. The development of effective vaccination strategies would therefore benefit from further insight into the mechanisms of successful LASV immune control, as well as into the processes underlying LF development and pathogenesis.

It is generally agreed upon that the level of tissue damage observed at autopsy cannot by itself account for the severe nature of LF. Therefore, as with other viral hemorrhagic fevers [6],[7], a contribution of the host response to LF pathogenesis has long been a matter of debate. For instance, the manifestation of Dengue Hemorrhagic Fever (DHF) has long been accredited to pre-existing immunity [8],[9]. Apart from serotype cross-reactive antibodies [8],[9], memory T cells were recently identified as important players in the disease process [10], and susceptibility as well as resistance to DHF have been linked to particular MHC alleles [11],[12]. In addition, infected monocytes and macrophages play an important role in DHF by secreting inflammatory cytokines [13],[14].

Such contributions of the immune response to disease severity can represent a major hurdle in vaccine development [15]. For instance, formalin-inactivated vaccines to respiratory syncytial virus (RSV) and measles virus resulted in enhanced morbidity and mortality in response to natural infection [16],[17]. Animal models for RSV have since provided evidence that T cell subsets play an important role in disease enhancement [16],[18]. Interestingly, innate immune cells including eosinophils and polymorphonuclear granulocytes dominate the histological picture upon T cell-driven enhancement of RSV [18]. Similarly, inflammatory macrophage responses were found to be a common feature of viral hemorrhagic fevers [6]. In accordance with the “cytokine storm” hypothesis, macrophage-derived inflammatory cytokines [19],[20],[21],[22],[23] and nitric oxide (NO) [24],[25] are candidate mediators of capillary leakage and shock [26], and elevated levels of such mediators correlate with increased disease severity and worsened clinical outcome.

Still, LASV lacks a clear pathognomonic signature, and clinical manifestations of LF are largely unspecific, making it difficult to diagnose the infection accurately via clinical criteria alone [27]. In contrast to other hemorrhagic fevers, coagulation abnormalities and bleeding are largely absent in LF [27],[28], leading some to argue on pathological grounds that Lassa fever ought not be considered a hemorrhagic fever at all [6],[29]. More characteristic of severe LF cases are the vascular leakage with edema and effusions in the pleural and pericardial cavities [30],[31],[32]. At necropsy, liver and lung count among the organs most commonly affected during LF [29],[30],[31],[32],[33].

One of the few well-documented characteristics of primary LASV-directed immune response is that neutralizing antibody responses develop only weeks or months after the virus has been eliminated [28]. Also studies of vaccination-induced LASV immunity point toward a cell-mediated mechanism at the frontline of antiviral defense [34],[35]. Still, this notion remains to be addressed and verified directly, and the responsible T cell subtypes to be characterized. Further, a potential disease-enhancing effect of T cell responses in LF has not yet been given sufficient consideration.

Although normal laboratory mouse strains develop acute disease of the central nervous system when infected with LASV intracerebrally [1],[28],[36], they remain resistant to the systemic disease so characteristic of human LF, irrespective of the route used to infect them. Research on LF has therefore been limited to the use of guinea pigs and non-human primates [28], complicating mechanistic studies on immunity and pathogenesis. Here we report on a series of experiments triggered by accidental observations of serious disease in LASV-infected humanized mice (HHD mice [37], C57BL/6 background). HHD mice are genetically engineered to express a human/mouse-chimeric HLA-A2.1 molecule instead of the murine MHC class I gene products and are widely used to identify human HLA-A2.1-restricted peptide epitopes. Stimulated by these unexpected results, we were able to identify T cell-dependence of LASV control, but also of LF pathogenesis. These findings, combined with the propensity of LASV to target monocyte/macrophage lineage cells *in vivo*, followed by T cell-dependent activation of this cell population, provide a novel concept for virus-host relationship and pathogenesis of LASV. We anticipate that such understanding may aid rational refinement of both vaccine-mediated prevention and treatment of LASV infection.

## Results

### MHC class I-dependent control of LASV infection

**Figure 1:**
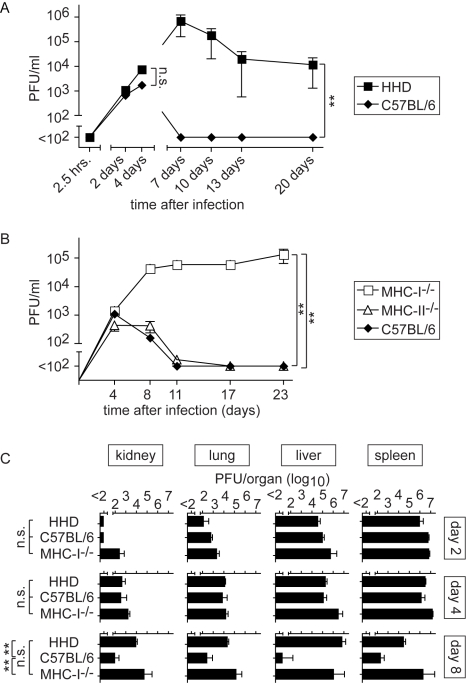
MHC-I but not MHC-II determines efficient LASV control. Mice of the indicated genotypes were infected with LASV. A, B: Blood samples were collected over time to determine viremia. A: The 2.5 hrs, 2 day and 4 day time points (left part of panel A) were determined in one experiment, and day 7, 10, 13 and 20 (right part of panel A) were determined in a second one. C: Mice were sacrificed on day 2, 4 and 8 after LASV infection to measure viral titers in organs. Axis breaks indicate the technical detection limit. Symbols and bars represent the mean±SEM of three to five mice per group.

We first compared viral replication in HHD mice and wild type C57BL/6 controls. C57BL/6 mice cleared LASV within about seven days after infection whereas HHD mice remained viremic for substantially longer periods of time (Fig. 1A and data not shown). A detailed analysis of the initial phase of infection documented a virtually immediate uptake of the inoculum into tissues (no virus in blood 2.5 hours after inoculation), followed by identical levels of viremia in wild type and HHD mice up to around day 4 (Fig. 1A). This demonstrates that viremia reflected viral replication in tissues rather than residual inoculum, and that the early phase of virus replication was identical in HHD and C57BL/6 mice. Clear differences in virus control became evident no earlier than seven days after infection (Fig. 1A). These differences in kinetics were compatible with differential adaptive immune control in HHD and C57BL/6 mice. Given that MHC class I (MHC-I) represents the only genetic difference between HHD and C57BL/6 mice, these findings suggested that H-2D^b^/H-2K^b^-restricted T cell responses in C57BL/6 mice played an important role in virus control. Hence, we extended our study to further analyze the contribution of MHC-I- and MHC-II-restricted T cell responses to LASV control (Fig. 1B). MHC-II-deficient mice (lacking CD4^+^ T cells) efficiently resolved the infection whereas MHC-I-deficient animals (MHC-I^-/-^; targeted mutation of the β2-microglobulin gene; devoid of CD8^+^ T cells) developed persistent high-level viremia. This corroborated the key role of MHC-I-restricted T cell responses in LASV control and indicated further that MHC-II-restricted responses were of lesser importance. A time course analysis of viral titers in kidney, lung, liver and spleen of LASV-infected HHD, C57BL/6 and MHC-I^-/-^ mice confirmed that viral replication was comparable on day 2 and day 4. By day 8, however, LASV in the organs of C57BL/6 mice approached the detection limit whereas comparably high titers of virus persisted in tissues of HHD and MHC-I^-/-^ mice. These data provided additional independent support for the above conclusions on productive replication of LASV in mice and the key role of MHC-I-restricted T cells in its control.

### T cell-dependent disease in LASV-infected HHD mice

**Figure 2:**
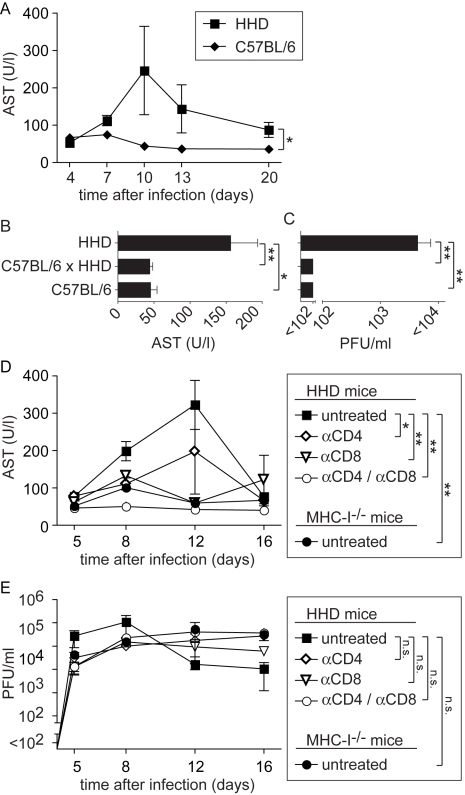
MHC-I- and T cell-dependent AST elevation suggest T cell-dependent immunopathogenesis of LF. A: C57BL/6 and HHD mice were infected with LASV. AST activity was determined in serum samples collected over time. B, C: In a separate experiment comprising C57BL/6, C57BL/6 x HHD F1 and HHD mice, serum AST activity (B) and viremia (C) were measured on day 8 after LASV infection. D, E: HHD mice were either left untreated, were depleted of CD8^+^ T cells or of CD4^+^ T cells or were depleted of both populations. All groups of HHD mice and MHC-I^-/-^ mice were subsequently infected with LASV. Serum AST activity (D) and viremia (E) was measured over time. Symbols and bars represent the mean±SEM of three to five mice per group and time point.

Between 7 and 12 days after LASV infection, HHD mice developed ruffled fur and reduced spontaneous activity. Some of them rapidly and unexpectedly deteriorated and progressed to a state of agony and death: Five out of twenty three mice (∼22%) infected in a total of five experiments succumbed to disease. In contrast but in accordance with the literature, all thirteen wild type C57BL/6 mice, serving as controls in three of these experiments, survived without clinical evidence of disease. Elevated serum aspartate aminotransferase activity (AST) represents the primary parameter for monitoring LF, and AST combined with viremia represent the best predictors for clinical outcome in humans [38]. In keeping with this manifestation of LF, serum AST activity remained mostly within normal ranges in wild type C57BL/6 controls but was significantly elevated in the serum of HHD mice, with a peak around day eight to twelve (Fig. 2A). To further investigate how T cell responses restricted to H-2K^b^/H-2D^b^ and to HLA-A2.1 influenced LASV control and disease, we crossed HHD mice to C57BL/6 mice. C57BL/6 x HHD F1 mice express H-2K^b^, H-2D^b^ and HLA-A2.1 molecules owing to hemizygosity at all relevant genetic loci. C57BL/6 x HHD F1 mice controlled LASV infection as efficiently as did C57BL/6 wild type mice, and their AST levels remained within normal ranges (Fig. 2B, C). This showed that H-2K^b^/H-2D^b^-mediated virus control prevented disease even in the presence of the HLA-A2.1 molecule. At first it suggested also that persistent and high virus load was directly responsible for pathogenesis. Contrary to this notion, however, the experiments in MHC-I-deficient mice had not resulted in obvious disease despite persistent high-level viremia. This raised the possibility that primary T cell responses may contribute to LF in an immunopathological fashion, similar to the role of memory T cell responses in DHF [10],[11],[39]. To address this possibility, we infected HHD mice with LASV, and prior to infection depleted either CD8^+^ T cells or CD4^+^ T cells or both using monoclonal antibodies. MHC-I^-/-^ mice are devoid of a CD8^+^ T cell compartment and were also included in the experiment. Unlike in untreated HHD mice and irrespective of comparably high levels of viremia in all groups (Fig. 2E), serum AST levels of CD8/CD4-double-depleted HHD mice remained in normal ranges (Fig. 2D). Also, depletion of only CD4^+^ or CD8^+^ T cells or genetic deficiency for MHC-I (affecting the CD8^+^ but not the CD4^+^ T cell compartment) afforded at least partial protection. In agreement with the results shown in Figs. 1B and 1C, these data suggested that T cells of HHD mice were unable to significantly influence viremia. Nevertheless CD8^+^ and CD4^+^ T cells played apparently an essential role in the pathogenesis of LF in HHD mice.

### Mopeia virus-induced T cell immunity protects HHD mice against Lassa fever

**Figure 3:**
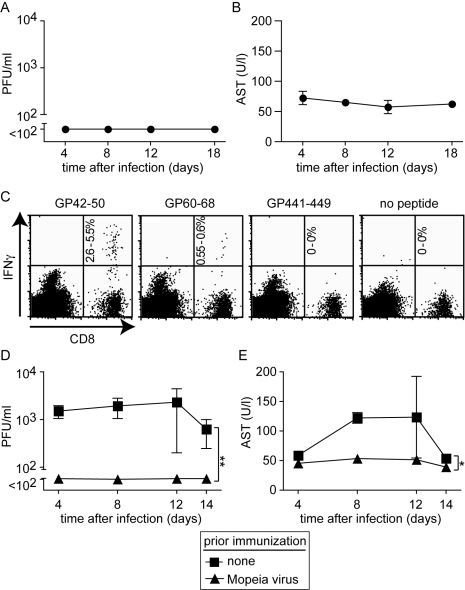
Mopeia virus (MV) infection is apathogenic in HHD mice but elicits LASV-specific HLA-A2.1-restricted CD8^+^ T cells and protects against subsequent LASV challenge. A, B: HHD mice were infected with MV. Serum and blood samples were collected at the indicated time points for measuring viremia (A) and AST (B), respectively. C: Epitope-specific CD8^+^ T cells in the spleen of MV-infected HHD mice were measured on day 8 (naive controls did not display detectable responses, data not shown). The percent range of IFNγ-producing CD8^+^ T cells is indicated in the upper right quadrant. D, E: HHD mice, either naive or infected with MV two weeks previously, were challenged with LASV. Viremia (D) and serum AST activity (E) were measured at the indicated time points. Symbols represent the mean ±SEM of four to five mice per group and time point.

To further characterize the role of T cells in the HHD mouse model for LF, we analyzed whether such animals could be immunized against LASV. For this, we used Mopeia virus (MV), an apathogenic close relative of LASV. MV infection is known to elicit heterologous immunity against LASV in monkeys (analogous to vaccinia virus protecting against smallpox), and MV and recombinants thereof have therefore been postulated as LASV vaccines [40],[41]. MV infection of HHD mice did not result in detectable viremia (Fig. 3A) nor was AST elevation recorded at any time point (Fig. 3B). This pattern of susceptibility of HHD mice to LASV but not MV reflected the one reported in non-human primates [40]. Next we tested whether MV immunization could induce HLA-A2.1-restricted immunity against LF. A recent study has characterized HLA-A2.1-restricted T cell epitopes in the glycoprotein (GP) of LASV [42]. Here we found that MV infection of HHD mice elicited high frequencies of CD8^+^ T cells specific for the GP42-50 epitope of LASV and a somewhat weaker but clearly detectable response against the GP60-68 epitope (Fig. 3C). CD8^+^ T cells specific for a third known epitope in LASV-GP (GP441-449) were not induced to a detectable extent, owing to only partial sequence homology of MV and LASV. When subsequently challenged with LASV, MV immunization prevented viremia and serum AST elevation in HHD mice (Fig. 3D, E), and by day 14 after challenge the spleen, liver, lung and kidney of MV-immunized mice were free of detectable LASV (data not shown). MV and LASV are serologically distinct i.e. neutralizing antibodies elicited against one virus do not crossreact nor crossprotect against the other [43]. This suggested that T cell immunity protected MV-immunized HHD mice against subsequent LASV challenge (Fig. 3D, E), albeit primary T cell responses facilitated apparently the disease process in unvaccinated animals (Fig. 2D).

### Histopathological alterations in diseased HHD mice are reminiscent of human Lassa fever and depend on T cells

**Figure 4:**
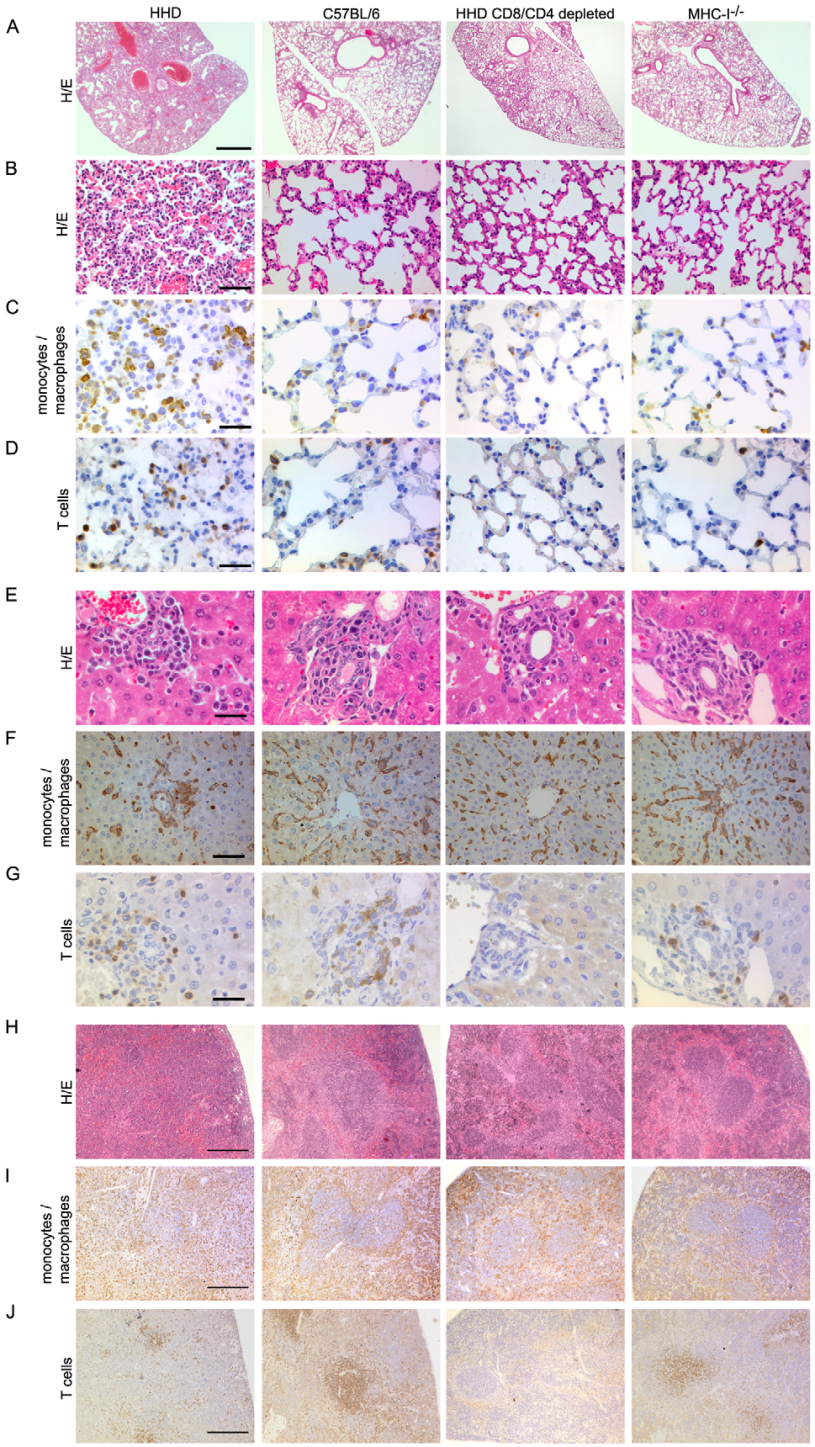
T cell- and MHC-I-dependent alterations in the tissues of LASV-infected mice. HHD, C57BL/6, HHD mice depleted of CD8^+^ and CD4^+^ T cells, and MHC-I^-/-^ mice were infected with LASV. Eight days later, lung (A-D), liver (E-G) and spleen (H-J) tissue was processed for histological analysis. H/E (A, B, E, H) or immunohistochemical staining of monocytes/macrophages (Iba-1; C, F, I) and T cells (CD3; D, G, J) are shown. Note the collapsed alveolar space, rounded and irregularly distributed monocytes/macrophages in liver and disruption of the splenic microarchitecture in LASV-infected HHD mice. These changes are less severe or largely restored in C57/BL/6 and CD8/CD4-depleted HHD mice, respectively, whereas MHC-I^-/-^ mice display an intermediate picture. Magnification bars indicate 1000 µm (A), 100 µm (B), 50 µm (C, D, F), 30 µm E,G) and 200 µm (H-J), respectively.

Next we set out to characterize tissue alterations in LASV-infected HHD mice and to study their dependence on T cells. In all LASV-infected HHD mice analyzed, the lung showed severe pneumonitis with interlobular septal thickening and collapse of the alveolar lumen (Fig. 4A, B). In addition, macroscopic analysis at necropsy or in terminally diseased animals often revealed a substantial pleural effusion (up to about 0.5 ml in each hemithorax, not shown). Both observations matched those in human LF [30],[31]. In contrast to HHD mice, the lungs of C57BL/6 sacrificed at the same time point were only mildly affected or appeared normal (Fig. 4A, B). CD8/CD4-depletion prevented these alterations in HHD mice, and also MHC-I^-/-^ mice exhibited considerably milder signs of peumonitis. Interestingly, HHD lungs contained dense infiltrations of rounded Iba-I^+^ monocyte/macrophage lineage cells (Fig. 4C), a finding that was less prominent or absent in C57BL/6 mice, T cell-depleted HHD mice or MHC-I^-/-^ mice. Accumulation of T cells was also noted in HHD lungs (Fig. 4D) albeit to a lesser extent than for monocytes/macrophages (compare Fig. 4C). Moreover, similar infiltrations were also found in resistant C57BL/6 wild type mice and thus did not correlate with disease.

In the liver, nodules of mononuclear cells were found around the portal fields of all four groups of mice (Fig. 4E). Striking differences were, however, noted in the distribution, shape and orientation of hepatic monocyte/macrophage populations (including Kupffer cells, Fig. 4F). Like in uninfected mice, hepatic monocytes/macrophages of C57BL/6 and of T cell-depleted HHD mice formed predominantly a flat layer along liver sinusoids, oriented towards the central vein in a stellar pattern. In contrast, the architecture of this cell layer was disrupted in HHD mice (unless depleted of T cells) with the remaining cells enlarged, rounded up, disorganized and often accumulated in clusters, indicative of cellular activation and reminiscent of the vigorous hepatic macrophage response reported from human LF [29],[30],[31],[32],[33]. MHC-I^-/-^ mice displayed an intermediate picture with only moderate monocyte/macrophage activation. Conversely, T cells were scattered at similarly moderate density throughout the liver parenchyma and in periportal inflammatory nodules of HHD and C57BL/6 mice (Fig. 4G and data not shown). The number of hepatic T cells did therefore not correlate with disease, similar to the findings in the lung.

Generalized immunosuppression is widely assumed to accentuate viral hemorrhagic fever [6]. A recent monkey study has tentatively attributed LASV immunosuppression to disorganization of the microarchitecture in secondary lymphoid organs [44]. Here we found that LASV infection of HHD mice resulted in disruption of the splenic white and red pulp compartments, whereas the spleens of C57BL/6, CD8/CD4-depleted HHD mice and MHC-I^-/-^ mice were less affected (Fig. 4H). In correlation with these alterations, the marginal zone macrophage layer was lost in HHD mice but not in the other groups, and monocytes/macrophages were homogenously distributed throughout the splenic tissue of HHD mice (Fig. 4I). T cells were largely absent in CD8/CD4-depleted mice, as expected, and were also somewhat scarce in HHD mice (Fig. 4J), possibly indicating LASV-induced T cell depletion as reported from non-human primates [44].

### Monocytes/macrophages represent a major target of LASV and produce IL-12p40 in a T cell-dependent fashion

**Figure 5:**
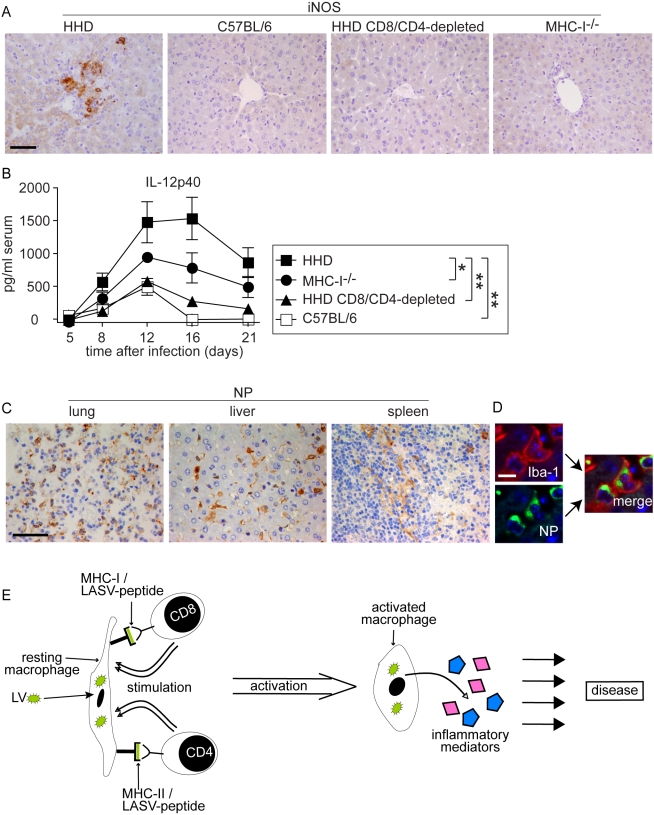
Monocytes/macrophages represent a major target of LASV and produce inflammatory mediators in a T cell-dependent fashion. A, B: HHD, C57BL/6, HHD mice depleted of CD8^+^ and CD4^+^ T cells, and MHC-I^-/-^ mice were infected with LASV. A: Eight days later, liver tissue was processed for histological analysis of iNOS expression. B. Serum samples were collected over time and IL-12p40 was determined by ELISA. C: Immunohistochemical staining of LASV-NP in lung, liver and spleen of HHD mice on day 8 after infection. D: Illustrative examples for cellular colocalization of the monocyte/macrophage marker Iba-1 (red) and LASV-NP (green) in the liver. Magnification bars indicate 50 µm (A, C) and 10 µm (D), respectively. E: Schematic describing the postulated mechanism how T cells may enhance LF pathogenesis.

The above morphological alterations had suggested T cell-dependent monocyte/macrophage activation in LASV-infected HHD mice. Classical activation of monocytes/macrophages e.g. by the T cell cytokine interferon gamma and cell-to-cell contact [45],[46],[47],[48] triggers the secretion of NO and inflammatory cytokines, and expression of the former is mediated by inducible NO synthase (iNOS) [45],[46],[47],[48],[49]. iNOS expression can therefore serve as a histological marker for inflammatory differentiation of monocytes/macrophages [50]. On day 8 after LASV infection we detected numerous iNOS-expressing monocyte/macrophage clusters in the liver parenchyma of HHD mice (Fig. 5A). Conversely, iNOS expression was not found in the liver of C57BL/6 mice, CD8/CD4-depleted HHD mice or MHC-I^-/-^ mice infected with LASV. Furthermore, neither HHD nor C57BL/6 mice displayed hepatic iNOS expression or morphological evidence of monocyte/macrophage activation when assessed on day 2 and day 4 after infection (Fig. S1, analogous data in lung and spleen not shown), i.e. prior to the onset of the adaptive immune response.

Synthesis of the inflammatory cytokine subunit IL-12p40 is restricted to macrophages, monocytes and dendritic cells, and its production is greatly enhanced by T cell stimulation [48],[51]. Within eight days after LASV infection, susceptible HHD mice displayed vastly elevated serum IL12-p40 levels (Fig. 5B). Resistant C57BL/6 mice showed comparably moderate IL-12p40 elevation. In agreement with the above results on iNOS, IL-12p40 secretion was strongly reduced by CD4/CD8-depletion in HHD mice, and MHC-I^-/-^ mice exhibited an intermediate IL-12p40 response. Together, these findings suggested that T cells triggered an inflammatory differentiation of monocytes/macrophages with subsequent production of NO, IL-12p40 and likely other inflammatory mediators (see Discussion section). This process occurred, however, solely under conditions of unchecked LASV replication i.e. in HHD mice but not in C57BL/6 mice where the infection and thus the antigen were rapidly cleared.

We had noted high virus loads in lung, liver and spleen of HHD mice (Fig. 1C) where the above pathological changes were found. The cellular distribution of LASV *in vivo* remains unknown, albeit the virus has been shown to replicate productively in cultured primate macrophages and dendritic cells [21],[52],[53]. To better understand how viral replication might be associated with disease we assessed virus distribution in tissues by immunohistochemistry on day eight after infection (Fig. 5C). LASV nucleoprotein (NP) was readily detected in a distinct population of cells in lung, liver and spleen. Morphological criteria suggested that these cells were predominantly monocytes/macrophages. We therefore performed immunofluorescence double-stains for LASV nucleoprotein (LASV-NP) and the monocyte/macrophage marker Iba-I (Fig. 5D). By this method, 82±6.8% of LASV-NP^+^ cells in liver, 65±8.5% in the lung and 79±11% in the spleen (mean±SD of six mice) could be identified as monocytes/macrophages, suggesting that they served as a major target of LASV.

## Discussion

The data in mice presented here suggest a dual role for T cells during LASV infection: T cells appear essential for rapid clearance of the virus, but if failing to do so they may play a key role in the ensuing disease process, too. Involvement of T cells in the pathogenesis of viral hemorrhagic fever has precedence in Dengue virus infection [10],[11],[39]. Unlike dengue hemorrhagic fever (DHF), where “original antigenic sin” of memory T cells appears to be involved, our findings suggest that T cell responses can have disease-enhancing effects during primary LASV infection. Importantly, we do not exclude roles for memory T cells in addition, but such aspects remain to be investigated (see below).

Our experiments modeled three prototypic scenarios of LASV-host balance, as defined by the parameters “T cells” (relative efficacy of antiviral T cell responses) and “Virus” (persistent virus load), thus delineating the extremes of a spectrum and resulting in the following outcomes:

- T cells^+^ Virus^−^: Rapid and efficient virus control by T cells prevents disease (naive C57BL/6 and vaccinated HHD mice).
- “T cells^+^ Virus^+^”: An antiviral T cell response of intermediate efficiency fails at eliminating the virus, but triggers severe disease (naive HHD mice).
- “T cells^−^ Virus^+^”: Complete absence of T cell defense allows LASV persistence, and results in only mild or absent disease (CD8/CD4-depleted HHD mice; partial protection in MHC-I^-/-^ mice).

Several mechanisms can be envisaged by which T cell responses enhance LF [10],[11],[39] but additional studies will be needed to address them directly. The histological picture in HHD mice supports the current view [7] that direct T cell-mediated cytolysis [54] unlikely is the main mechanism responsible for the tissue damage in LF, such as hepatocyte death with subsequent AST release. Based on the available evidence we postulate that during persisting viremia, T cells continuously encounter LASV epitopes on infected monocytes/macrophages in MHC-I and MHC-II context (Fig. 5E). Additional interaction via co-stimulatory molecules, or stimulation via T cell cytokines may trigger infected monocytes/macrophages to differentiate and subsequently secrete inflammatory mediators of their own [45],[48],[49],[51]. Overstimulation of macrophages can result in severe hepatic and pulmonary damage besides mediating a shock syndrome [26],[55],[56], and such overstimulation provides a plausible mechanism for indirect T cell involvement in LF pathogenesis. LASV and related viruses are known to replicate in cultured macrophages without causing cellular activation or production of inflammatory cytokines [21],[52],[53]. Hence, classical T cell-driven monocyte/macrophage activation by IFNγ and direct cell-to-cell contact [45],[46],[47],[48] may augment inflammatory differentiation and cytokine release from LASV-infected monocytes/macrophages *in vivo* [19],[21],[22], similar to the ability of LPS to induce the activation of LASV-infected macrophages in culture [21],[52],[53]. T cell stimulation may thus facilitate a systemic inflammatory condition [57] as a potential pathogenetic correlate of the diverse and non-specific clinical manifestations of LF.

This view of the role of T cells in LF correlates well with our observations in scenario I, where one would predict that efficient virus elimination by C57BL/6 and immune HHD mice results in only short and transient antigen presentation on a limited number of infected monocytes/macrophages, and therewith lack of disease, as was indeed found. Similarly, the concept explains our findings in scenario III, where the absence of T cells to properly activate infected monocytes/macrophages in CD8/CD4-depleted HHD mice results in mild or absent disease.

In addition, at least two – seemingly paradoxical – observations in LASV-infected non-human primates would support our mechanistic postulate. The first observation is that high doses of LASV tend to be less lethal than low ones [28]. This phenomenon may be explained through the mechanisms of T cell “exhaustion” or “deletion” under high virus loads [58]. As such, a high initial virus inoculum may weaken the T cell response, thus attenuating disease through shifting conditions from scenario II towards scenario III.

The second paradoxical observation stems from a vaccination study, in which a recombinant vaccinia virus expressing the NP protein of LASV was used in monkeys. The vaccine turned out to protect only a minority of the animals and, intriguingly, those LASV-challenged monkeys not protected by the vaccine displayed a more acute form of disease than control monkeys that had not been vaccinated at all [34]. A likely explanation may be that, although the vaccination may not have protected all animals, the still accelerated (memory) T cell response of non-protected animals also accelerated their disease process. Support for such a scenario comes from infection of mice with another arenavirus, lymphocytic choriomeningitis virus (LCMV). LCMV-induced immunopathological disease of the central nervous system is T cell-dependent and, similar to the observation with LASV in monkeys, can be enhanced by prior vaccination with recombinant vaccinia viruses expressing LCMV antigens [59].

Last but not least, our study introduces a mouse model for LF, the lack of which has long hampered progress in this field of research. Only the general versatility of the mouse as a research model, including the availability of gene-targeted strains, makes mechanistic studies as presented here possible. Despite certain shortcomings as listed below, we think that the humanized mouse model could prove useful in further studies on LF pathogenesis, especially as the model lends itself well to the assessment of CD8^+^ T cell-based vaccines (compare Fig. 3). Although the ∼20% lethality we found in HHD mice using the Ba366 strain of LASV may contrast with the uniform lethality observed in LASV strain Josiah-infected strain 13 guinea pigs or monkeys [28], our lethality rates match those reported for Josiah-infected outbred Hartley guinea pigs [60], as well as inbred strain 13 and strain 2 guinea pigs inoculated with other LASV isolates [61]. Nevertheless, we cannot exclude the possibility that the HHD model fails to recreate certain aspects of human LF.

For the mechanistic analyses presented here, we used relatively high intravenous LASV doses of 10^6^ PFU. The need for such doses to cause severe disease likely reflects the imperfect adaptation of LASV to mice (compare also the Methods section). However, preliminary experiments indicate that viremia and AST elevation can already be observed at lower doses, albeit with higher variability, and that LASV can replicate in HHD mice after subcutaneous administration (data not shown). We would therefore argue that viremia and AST elevation in HHD mice may represent useful surrogates [38] to assess vaccine efficacy prior to an eventual confirmation in non-human primates.

T cell densities in the spleen of LASV-infected HHD and C57BL/6 mice were comparable (day 8: 3339±925 CD3^+^ cells/mm^2^ in HHD mice, 3826±2057 CD3^+^ cells/mm^2^ in C57BL/6 mice; mean±SD, n = 6; p = 0.61), arguing against preferential T cell depletion [44] as a potential reason for defective virus control in HHD mice. To characterize disease enhancing (HHD) vs. protective (C57BL/6) primary T cell responses against LASV, future studies will need to compare the magnitude, kinetics and effector-/cytokine-profile [62] of LASV epitope-specific CD4^+^ and CD8^+^ T cell responses. Similarly, serum IL-12p40 and iNOS expression have served as surrogates of the inflammatory monocyte/macrophage response in this study, but future work will have to determine its breadth in terms of cytokines, chemokines and inflammatory mediators such as NO, leukotrienes and prostaglandins, their production in tissues and systemic dissemination in blood. The experimental depletion and/or inhibition of monocyte/macrophage populations and of inflammatory mediators may provide additional insights into the cellular and molecular players in LF, indicating to which extent the “cytokine storm” hypothesis [57] can explain LF pathogenesis. With the availability of a mouse model for LASV such studies become possible, although the necessity for BSL-4 laboratory containment can represent a major practical hurdle.

Taken together, our results in mice suggest a two faced role of T cells in LASV infection, both in virus control and also in enhancing LF pathogenesis. This extends our understanding of LASV-host interactions and raises the possibility that heterogeneity in MHC-I and in overall T cell immunocompetence represents one explanation for the wide spectrum of clinical outcomes in a human population exposed to LASV [2]. Perhaps even more important, we think that beside beneficial also detrimental aspects of T cell-responses and -immunity [34],[59] should be given thorough consideration in future strategies for LF vaccine design.

## Materials and Methods

### Mice and animal experiments

C57BL/6, β2-microglobulin-deficient mice (MHC-I^-/-^) [63], MHC-II^-/-^ [64] and HHD [37] mice were bred at the Institute for Laboratory Animal Sciences, University of Zurich, Switzerland. Experiments with Lassa virus were performed in the BSL-4 unit of the Bernhard Nocht Institute, Hamburg, Germany. Experiments with Mopeia virus were performed at the University of Geneva and at the University Hospital of Zurich, Switzerland. Permission for animal experiments was obtained from the authorities of the Freie und Hansestadt Hamburg, and from the Cantonal authorities of Geneva and Zurich, Switzerland, respectively. All experiments were performed in accordance with the Swiss and German law for animal protection, respectively.

### Viruses, virus titration, infection and T cell depletion of mice

This model is based on the Ba366 [65] strain of LASV. Pilot experiments with a range of isolates representing the different endemic areas (Josiah, Sierra Leone; Lib90, Liberia; Ba366, Guinea; AV, Ivory coast/Burkina Faso; CSF, Nigeria) had indicated that Ba366 was the virus that most efficiently replicated in HHD mice. LASV and Mopeia virus (AN21366), were grown on BHK21 and Vero cells, respectively, and were administered to mice at a dose of 10^6^ PFU i.v. unless stated differently. Virus stocks and viral infectivity in blood samples were determined in immunofocus assays as described [66]. CD8^+^ and/or CD4^+^ T cell populations were depleted by i.p. administration of monoclonal antibodies YTS169 (anti-CD8) and YTS191 (anti-CD4) on day -3 and day -1 of LASV infection as previously described [67]. The efficiency of depletion was verified by flow cytometry and was >99%.

### Blood biochemistry and histology

Serum AST and ALT activities were determined by using commercially available colorimetric assay kits (Reflotron, Roche Diagnostics, Germany). Mouse tissues were fixed in 4% formalin and were embedded in paraffin. Sections were stained with hematoxilin/eosin (H/E) or processed for immunohistochemistry as follows: Upon inactivation of endogenous peroxidases (PBS/3% hydrogen peroxide) and blocking (PBS/10% FCS) sections were incubated with the primary antibodies rat anti-human CD3 (crossreactive with murine CD3 on mouse T cells; Serotec), rabbit anti-Iba-1 (monocytes/macrophages, Wako Pure Chemical Industries) or rat anti-Lassa nucleoprotein (see below). Bound primary antibodies were detected with biotinylated rat-specific (DakoCytomation) or rabbit-specific (Amersham) secondary antibody, followed by incubation with extraavidin peroxidase (Sigma Aldrich), and bound peroxidase was visualized by 3,3′-diaminobenzidine as chromogen (Sigma Aldrich). Haemalaun was used for counterstaining of nuclei. For fluorescence double labeling, primary antibodies were visualized using species specific Cy3- or Cy2-conjugated secondary antibodies (all from Jackson ImmunoResearch Laboratories Inc.) with DAPI (Sigma-Aldrich) nuclear staining. To determine the percentage of monocytes/macrophages (Iba-1-positive cells) amongst LASV-infected cells, a total of 41 (liver) and 27 (lung) randomly captured 40x visual fields were analyzed.

### Automated assessment of CD3^+^ T cell densities in spleen sections

Histological spleen sections stained with anti-CD3 antibody (T cells) and counterstained with Haemalaun (nuclei, see above) were scanned using the Dotslide System (Olympus GmBH) at a 200-fold magnification. For analysis, the images were automatically processed in a custom-programmed script of Cognition Network Language based on the Definiens Cognition Network Technology platform (Definiens Developer XD software). The Cognition Network Language is an object-based procedural computer language, designed for automated analysis of complex, context-dependent image analysis. In brief, the programmed script first discriminates spleen tissue and tissue-free surroundings by spectral difference detection. The surface of the resulting region of interest (spleen tissue, ROI) is calculated. Subsequently “CD3 positive cells” within the ROI are detected based on brown anti-CD3 staining and are counted, to calculate the number of CD3^+^ cells per mm^2^ of tissue.

### ELISA

Serum IL-12p40 was determined using a sandwich ELISA kit (eBioscience) according to the manufacturer's instructions.

### Generation of anti-LASV-NP serum

Recombinant NP of LASV strain BA366 was expressed in E. coli using the pET28 expression vector system (Novagen). Supernatants of pET28 constructs were purified using the Talon Metal Affinity Resin (Clontech) in a batch procedure. Urea (8 M) lysates were brought to nondenaturing conditions by increasingly substituting the buffer for sonication buffer during the resin-batch procedure. Proteins were eluted with 250 mM imidazole in sonication buffer on a gravity column (Bio-Rad). Rat antisera were raised against purified recombinant NP by s.c. immunization with recombinant NP emulsified in complete Freund's adjuvant containing 1 mg of Mycobacterium tuberculosis (H37RA; Difco Laboratories, Detroit, MI). Four weeks after the first immunization, animals were boosted with recombinant NP emulsified in incomplete Freund's adjuvant (Difco). Terminal bleedings were performed 4 weeks after the boost. The specificity of the anti-LASV-NP antiserum was verified by immunofluorescence tests on LASV-infected cells as well as on LASV-infected or non-infected tissues. Pre-immune serum from the rats used for immunization was included as a control in both settings.

### Intracellular cytokine assay

Epitope-specific CD8^+^ T cells were enumerated by an intracellular cytokine assay for IFNγ as previously described [68]. In brief, 10^6^ splenocytes were incubated in 200 µl of IMDM supplemented with 10% FCS and penicillin/streptomycin for 5 h at 37°C at a 10^−6^ M concentration of the LASV-GP-derived peptide epitope GP42-50 (GLVGLVTFL), GP60-68 (SLYKGVYEL), GP441-449 (YLISIFLHL) or with medium alone as a negative control. To enhance intracellular accumulation of IFN-γ, brefeldin A was added at a final concentration of 5 µg/ml for the last 3.5 hours of culture. Subsequently, the cells were washed with FACS buffer (PBS supplemented with 2% FCS, 0.01% NaN_3_ and 20 mM EDTA) and surface staining was performed with anti-CD8β-PE and anti-B220-PerCP antibody conjugates (both from BD Biosciences) for 30 min at 4°C. After washing twice with FACS buffer, the cells were fixed with 100 µl of 4% paraformaldehyde in PBS for 5 min at 4°C. Two milliliters of permeabilization buffer (FACS buffer supplemented with 0.1% w/v saponin, Sigma) were added and the cells were incubated for 10 min at 4°C. Subsequently, they were spun down and stained intracellularly with anti-mouse-IFNγ-APC (BD Biosciences) in permeabilisation buffer for 60 min at 4°C. After two washes with permeabilization buffer, the cells were resuspended in FACS buffer and were analyzed on a FacsCalibur (Becton Dickinson). FACS plots were gated on B220^−^ lymphocytes.

### Statistical analysis

Between group differences were analyzed by 1-way ANOVA and 2-way ANOVA for individual or multiple values of different groups, respectively, followed by LSD post tests. SPSS vs. 13 was used for analysis. P values <0.05 were considered statistically significant (indicated as * in figures). P<0.01 was considered highly significant (indicated as ** in figures). P>0.05 was considered as not significantly different (“n.s.”).

## Supporting Information

Figure S1 Absence of detectable macrophage activation in the early phase of LASV infection. HHD and C57BL/6 mice were infected with LASV. Two, four and eight days later, liver tissue was processed for histological analysis. Tissues of uninfected mice served as reference. H/E staining and immunohistochemical detection of monocytes/macrophages (Iba-1) and iNOS are shown. Arrowheads point out clusters of iNOS-positive monocytes/macrophages, which are found in day 8 LASV-infected HHD mice only. Magnification bars indicate 50 µm (H/E) and 100 µm (monocytes/macrophages and iNOS), respectively. Representative images from three mice per group and time point are shown.
(11.44 MB TIF)
