# Supplementary figures and images for: T Cell-Dependence of Lassa Fever Pathogenesis
Source: PLoS Pathog. 2010 Mar 26;6(3):e1000836. doi: 10.1371/journal.ppat.1000836 (PMC2847900; doi:10.1371/journal.ppat.1000836)

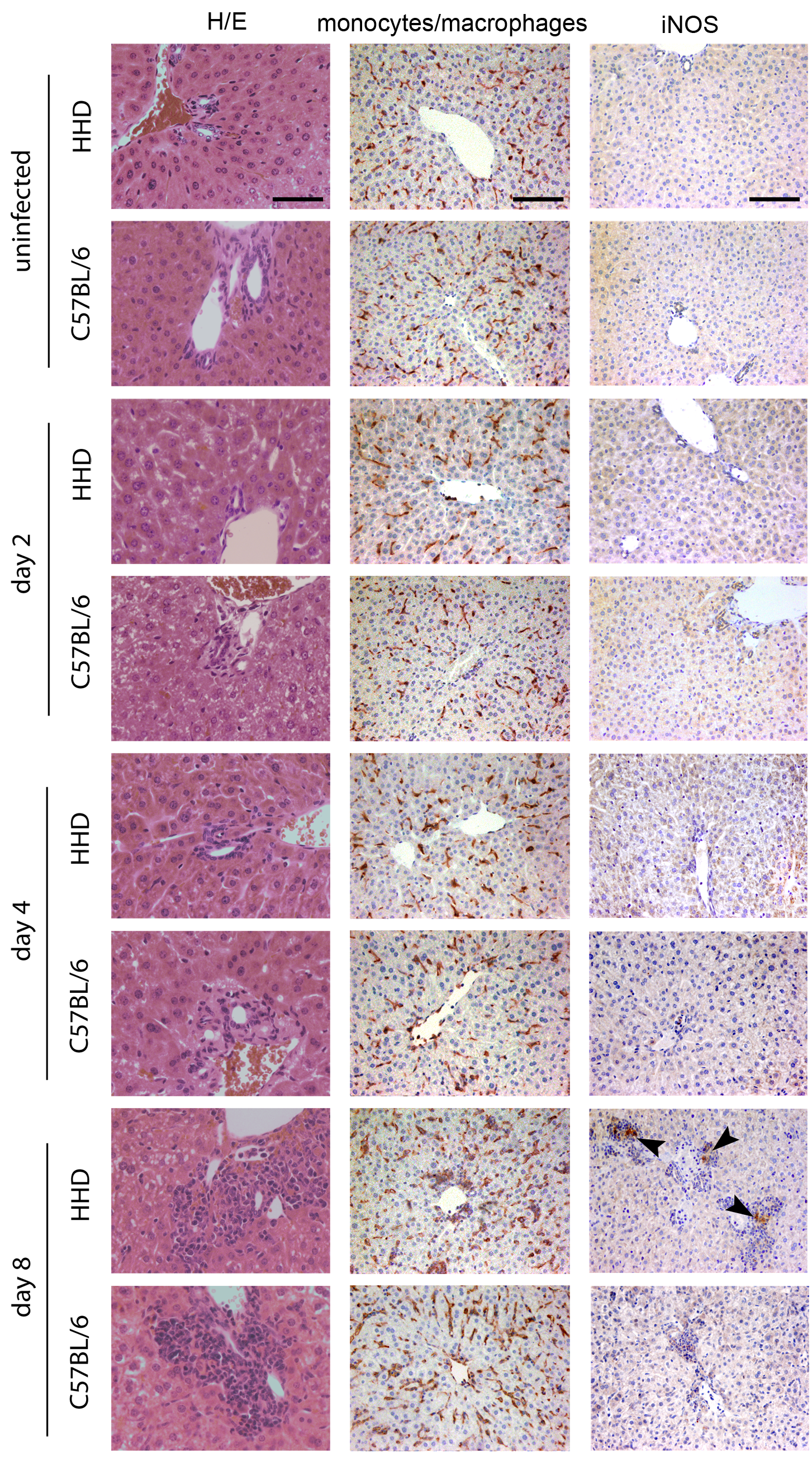

Supplement: Figure S1 — Absence of detectable macrophage activation in the early phase of LASV infection. HHD and C57BL/6 mice were infected with LASV. Two, four and eight days later, liver tissue was processed for histological analysis. Tissues of uninfected mice served as reference. H/E staining and immunohistochemical detection of monocytes/macrophages (Iba-1) and iNOS are shown. Arrowheads point out clusters of iNOS-positive monocytes/macrophages, which are found in day 8 LASV-infected HHD mice only. Magnification bars indicate 50 µm (H/E) and 100 µm (monocytes/macrophages and iNOS), respectively. Representative images from three mice per group and time point are shown. (11.44 MB TIF) [file ppat.1000836.s001.tif]
